# Supplementary material for: Cumulative SARS-CoV-2 mutations and corresponding changes in immunity in an immunocompromised patient indicate viral evolution within the host
Source: Nat Commun. 2022 May 10;13:2560. doi: 10.1038/s41467-022-30163-4 (PMC9090742; doi:10.1038/s41467-022-30163-4)
Supplement: Supplementary file 2 — Reporting Summary [file 41467_2022_30163_MOESM2_ESM.pdf]

nature portfolio

Corresponding author(s): Sissy T. Sonnleitner

Last updated by author(s): Apr 5, 2022

Reporting Summary

Nature Portfolio wishes to improve the reproducibility of the work that we publish. This form provides structure for consistency and transparency in reporting. For further information on Nature Portfolio policies, see our Editorial Policies and the Editorial Policy Checklist.

Statistics

For all statistical analyses, confirm that the following items are present in the figure legend, table legend, main text, or Methods section.

n/a	Confirmed	
☐	☒	The exact sample size (*n*) for each experimental group/condition, given as a discrete number and unit of measurement
☐	☒	A statement on whether measurements were taken from distinct samples or whether the same sample was measured repeatedly
☐	☒	The statistical test(s) used AND whether they are one- or two-sided<br>*Only common tests should be described solely by name; describe more complex techniques in the Methods section.*
☐	☒	A description of all covariates tested
☐	☒	A description of any assumptions or corrections, such as tests of normality and adjustment for multiple comparisons
☐	☒	A full description of the statistical parameters including central tendency (e.g. means) or other basic estimates (e.g. regression coefficient) AND variation (e.g. standard deviation) or associated estimates of uncertainty (e.g. confidence intervals)
☐	☒	For null hypothesis testing, the test statistic (e.g. F, t, r) with confidence intervals, effect sizes, degrees of freedom and P value noted<br>*Give P values as exact values whenever suitable.*
☐	☒	For Bayesian analysis, information on the choice of priors and Markov chain Monte Carlo settings
☐	☒	For hierarchical and complex designs, identification of the appropriate level for tests and full reporting of outcomes
☒	☐	Estimates of effect sizes (e.g. Cohen's *d*, Pearson's *r*), indicating how they were calculated

Our web collection on statistics for biologists contains articles on many of the points above.

Software and code

Policy information about availability of computer code

Data collection	Ion TorrentTM S5 Plus. Ion Torrent Suite software (v 5.12.2)
Data analysis	Unipro UGENE v.36; MEGA X.64; Pangolin software, v.2.4.2. (https://pangolin.cog-uk.io/); GISAID EpiCoV (https://gisaid.org/no.EPI_ISL_2106191-21061201); PhyloSuite v.1.2.2.

For manuscripts utilizing custom algorithms or software that are central to the research but not yet described in published literature, software must be made available to editors and reviewers. We strongly encourage code deposition in a community repository (e.g. GitHub). See the Nature Portfolio guidelines for submitting code & software for further information.

Data

Policy information about availability of data

All manuscripts must include a data availability statement. This statement should provide the following information, where applicable:

- Accession codes, unique identifiers, or web links for publicly available datasets
- A description of any restrictions on data availability
- For clinical datasets or third party data, please ensure that the statement adheres to our policy

The generated full-genome sequences are available at GISAID EpiCoV (http:/gisaid.org/), no. EPI_ISL IDs: 2106191 [https://www.epicov.org/epi3/frontend#2870a], 2106192 [https://www.epicov.org/epi3/frontend#590381], 2106193 [https://www.epicov.org/epi3/frontend#3535ef], 2106194 [https://www.epicov.org/epi3/frontend#2db57f], 2106195 [https://www.epicov.org/epi3/frontend#217409], 2106196 [https://www.epicov.org/epi3/frontend#10c3a8], 2106197 [https://www.epicov.org/epi3/frontend#22f37a], 21061200 [https://www.epicov.org/epi3/frontend#4148ed] and 21061201 [https://www.epicov.org/epi3/frontend#2893a7] in .fasta format and at Genome Sequencing Archives as .bam files under the Submission ID subCRA009634, Accession numbers CRR453213 (day 73), CRR453214 (day 93), CRR453215 (day 117), CRR4536216 (day 123), CRR453217 (day 129), CRR453218 (day 136), CRR453219 (day 143), CRR453220 (day 158),

CRR453221 (day 164), CRR453222 (day 171), CRR453223 (day 182). Source data are provided with this paper. Source data are provided with this paper.

Field-specific reporting

Please select the one below that is the best fit for your research. If you are not sure, read the appropriate sections before making your selection.

☐ Life sciences ☐ Behavioural & social sciences ☒ Ecological, evolutionary & environmental sciences

For a reference copy of the document with all sections, see nature.com/documents/nr-reporting-summary-flat.pdf

Life sciences study design

All studies must disclose on these points even when the disclosure is negative.

Sample size	not necessary
Data exclusions	not necessary
Replication	not necessary
Randomization	not necessary
Blinding	not necessary

Behavioural & social sciences study design

All studies must disclose on these points even when the disclosure is negative.

Study description	not necessary
Research sample	not necessary
Sampling strategy	not necessary
Data collection	not necessary
Timing	not necessary
Data exclusions	not necessary
Non-participation	not necessary
Randomization	not necessary

Ecological, evolutionary & environmental sciences study design

All studies must disclose on these points even when the disclosure is negative.

Study description	The study investigated the evolution of a SARS-CoV-2 strain in an immunocompromised patient during prolonged infection.
Research sample	Nasopharyngeal swabs were obtained on day 73, 93, 109, 129, 133, 136, 143, 158, 164, 171, 182, 192 and day 207 of the patient´s prolonged infection in the course of medical checks.
Sampling strategy	Nasopharyngeal swabs were obtained on day 73, 93, 109, 129, 133, 136, 143, 158, 164, 171, 182, 192 and day 207 of the patient´s prolonged infection in the course of medical checks.
Data collection	The nasopharyngeal swabs were taken from Nov20 - June21 during routine medical treatments.
Timing and spatial scale	According to Austrian guidelines, a SARS-CoV-2 swab was taken from the patient each time she was hospitalised for medical treatment of their underlying disease, as well as weekly for routine monitoring.
Data exclusions	Low quality sequences were excluded. Quality criteria were: Mapped reads > 1 mio., mean read length: >180 bp; mean depth >10,000.
Reproducibility	all sequences were confirmend by an independent sequencing group (GenXPro Germany). All nasopharyngeal sampes were sent to the independent study group, were extracted, sequenced and analysed there using their own methods. The second study group

	confirmed all our study results.
Randomization	allocation was not relevant for our study
Blinding	In our study, there were no study participants to be blinded, but the investigation of repositories from routine diagnostics. But the second, independent study group, was blinded in that form, that they did not know our results and, nevertheless, found the same.

Did the study involve field work? ☐ Yes ☒ No

Field work, collection and transport

Field conditions	not necessary
Location	not necessary
Access & import/export	not necessary
Disturbance	not necessary

Reporting for specific materials, systems and methods

We require information from authors about some types of materials, experimental systems and methods used in many studies. Here, indicate whether each material, system or method listed is relevant to your study. If you are not sure if a list item applies to your research, read the appropriate section before selecting a response.

Materials & experimental systems

n/a	Involved in the study	
☒	☐	Antibodies
☐	☒	Eukaryotic cell lines
☒	☐	Palaeontology and archaeology
☒	☐	Animals and other organisms
☐	☒	Human research participants
☐	☒	Clinical data
☒	☐	Dual use research of concern

Methods

n/a	Involved in the study	
☒	☐	ChIP-seq
☒	☐	Flow cytometry
☒	☐	MRI-based neuroimaging

Antibodies

Antibodies used	not necessary
Validation	not necessary

Eukaryotic cell lines

Policy information about cell lines

Cell line source(s)	Vero B4, DSMZ
Authentication	DSMZ ACC-33; Species-level identification was carried out by mitochondrial Cytochrome C Oxidase Subunit 1 (COI) DNA barcoding according standard ANSI/ATCC ASN-0003-2015 and revealed Chlorocebus aethiops
Mycoplasma contamination	The cell lines were not tested for Mycoplasma contanimation.
Commonly misidentified lines (See ICLAC register)	none

Palaeontology and Archaeology

Specimen provenance	not necessary
Specimen deposition	not necessary
Dating methods	not necessary

☐ Tick this box to confirm that the raw and calibrated dates are available in the paper or in Supplementary Information.

Ethics oversight	not necessary

Note that full information on the approval of the study protocol must also be provided in the manuscript.

Animals and other organisms

Policy information about studies involving animals; ARRIVE guidelines recommended for reporting animal research

Laboratory animals	not necessary
Wild animals	not necessary
Field-collected samples	not necessary
Ethics oversight	not necessary

Note that full information on the approval of the study protocol must also be provided in the manuscript.

Human research participants

Policy information about studies involving human research participants

Population characteristics	female patient in her sixties (65 years), diagnosed with stage IVa small cell lymphocytic lymphoma, six cycles of Rituximab and Bendamustine; three years later, the patient suffered a relapse with washout and 90% bone marrow infiltration (B-CLL Binet B or RAI III), accompanied by pronounced symptoms and antibody deficiency. Beginning in May 2020, another round of therapy with Rituximab and Bendamustine was administered. It was completed in November 2020 after six cycles. At that time, the leukocyte count was in the lower normal range at 4,200/µL, platelets 136,000/µL, the immunoglobulins were clearly reduced (IgG 249 mg/dL, IgA 3 mg/dL, IgM 12 mg/dL). Four days after the last chemotherapy the patient was tested positive for SARS-CoV-2 and stayed positive for the netx 7 months. in this time, nasopharyngeal swabs were taken routinely to evaluate the viral load. These swabs were taken for sequencing analysis.
Recruitment	The nasopharyngeal swabs were sent to the routine diagnostic lab for SARS-CoV-2 specific RT-PCR as well as mutation screening via whole genome sequencing. Also serologic samples were sent to the laboratory to investigate the patient´s specific immune response in the course of the patient´s individual medical treatment.
Ethics oversight	Ethical approval to use residual routinely taken serum samples for retrospective analyses was obtained by the Ethics Committee of the University Hospital Wuerzburg (no. 20201105_01).

Note that full information on the approval of the study protocol must also be provided in the manuscript.

Clinical data

Policy information about clinical studies

All manuscripts should comply with the ICMJEguidelines for publication of clinical research and a completedCONSORT checklist must be included with all submissions.

Clinical trial registration	not necessary
Study protocol	not necessary
Data collection	not necessary
Outcomes	not necessary

Dual use research of concern

Policy information about dual use research of concern

Hazards

Could the accidental, deliberate or reckless misuse of agents or technologies generated in the work, or the application of information presented in the manuscript, pose a threat to:

No	Yes	
☐	☒	Public health
☒	☐	National security
☒	☐	Crops and/or livestock
☒	☐	Ecosystems
☒	☐	Any other significant area

Hazards	*Please describe the agents/technologies/information that may pose a threat, including any agents subject to oversight for dual use research of concern.*

For examples of agents subject to oversight, see the United States Government Policy for Institutional Oversight of Life Sciences Dual Use Research of Concern .

Experiments of concern

Does the work involve any of these experiments of concern:

No	Yes	
☒	☐	Demonstrate how to render a vaccine ineffective
☒	☐	Confer resistance to therapeutically useful antibiotics or antiviral agents
☒	☐	Enhance the virulence of a pathogen or render a nonpathogen virulent
☒	☐	Increase transmissibility of a pathogen
☒	☐	Alter the host range of a pathogen
☒	☐	Enable evasion of diagnostic/detection modalities
☒	☐	Enable the weaponization of a biological agent or toxin
☒	☐	Any other potentially harmful combination of experiments and agents

Precautions and benefits

Biosecurity precautions	*Describe the precautions that were taken during the design and conduct of this research, or will be required in the communication and application of the research, to minimise biosecurity risks. These may include bio-containment facilities, changes to the study design/ methodology or redaction of details from the manuscript.*
Biosecurity oversight	*Describe any evaluations and oversight of biosecurity risks of this work that you have received from people or organizations outside of your immediate team.*
Benefits	*Describe the benefits that application or use of this work could bring, including benefits that may mitigate risks to public health, national security, or the health of crops, livestock or the environment.*
Communication benefits	*Describe whether the benefits of communicating this information outweigh the risks, and if so, how.*

ChIP-seq

Data deposition

☒ Confirm that both raw and final processed data have been deposited in a public database such as GEO.

☒ Confirm that you have deposited or provided access to graph files (e.g. BED files) for the called peaks.

Data access links *May remain private before publication.*	not necessary
Files in database submission	not necessary
Genome browser session (e.g. UCSC)	not necessary

Methodology

Replicates	not necessary
Sequencing depth	not necessary
Antibodies	not necessary
Peak calling parameters	not necessary
Data quality	not necessary
Software	not necessary

Flow Cytometry

Plots

Confirm that:

- [ ] The axis labels state the marker and fluorochrome used (e.g. CD4-FITC).
- [ ] The axis scales are clearly visible. Include numbers along axes only for bottom left plot of group (a 'group' is an analysis of identical markers).
- [ ] All plots are contour plots with outliers or pseudocolor plots.
- [ ] A numerical value for number of cells or percentage (with statistics) is provided.

Methodology

Sample preparation	not necessary
Instrument	not necessary
Software	not necessary
Cell population abundance	not necessary
Gating strategy	not necessary

- [ ] Tick this box to confirm that a figure exemplifying the gating strategy is provided in the Supplementary Information.

Magnetic resonance imaging

Experimental design

Design type	not necessary
Design specifications	not necessary
Behavioral performance measures	not necessary

Acquisition

Imaging type(s)	not necessary
Field strength	not necessary
Sequence & imaging parameters	not necessary
Area of acquisition	not necessary

Diffusion MRI ☐ Used ☐ Not used

Preprocessing

Preprocessing software	not necessary
Normalization	not necessary
Normalization template	not necessary
Noise and artifact removal	not necessary
Volume censoring	not necessary

Statistical modeling & inference

Model type and settings	not necessary
Effect(s) tested	not necessary

Specify type of analysis: ☐ Whole brain ☐ ROI-based ☐ Both

Statistic type for inference (See Eklund et al. 2016) | not necessary

Correction | not necessary

Models & analysis

n/a | Involved in the study
- [ ] [ ] Functional and/or effective connectivity
- [ ] [ ] Graph analysis
- [ ] [ ] Multivariate modeling or predictive analysis

Functional and/or effective connectivity | not necessary

Graph analysis | not necessary

Multivariate modeling and predictive analysis | not necessary
